# Use of healthcare services and hospitalisation among elderly people in Latvia

**DOI:** 10.1093/eurpub/ckac131.298

**Published:** 2022-10-25

**Authors:** S Dātava, I Reine

**Affiliations:** 1 Public Health, Riga Stradiņš University, Rīga, Latvia; 2 Statistic Unit, Riga Stradiņš University, Rīga, Latvia

## Abstract

**Background:**

Demographic changes have occurred worldwide during the recent decades, with an increasing proportion of the elderly in the society, leading to higher average age of the population. Older people are more likely to suffer from different chronic diseases, and higher consumption of healthcare services and, thus, posing additional burden and challenges to the healthcare system.

**Methods:**

The descriptive analysis relied on the longitudinal study of Health, Ageing and Retirement (SHARE). The target population included people aged 50 years or older, a total of 782 residents living in Latvia. We used the data from wave 8, collected from 2019-2020.

**Results:**

The mean age in the study population was 69 years, and 62.5 % of the respondents were women. Results show that 14.7 % of all respondents were hospitalised in the last 12 months. The average length of stay at hospital was 10.9 days (range 1 - 71, SD 10.5). In 36.7 % of the cases hospitalisation was planned, but in 42.9 % it was due to an emergency. More than a half had visited hospital only once, but 19.1 % four times or more. The most common diseases among the hospitalised patients were high blood pressure/hypertension (60 %), heart attack (27.8 %), high cholesterol (26.1 %), osteoarthritis (21.7 %), stroke (18.3 %), diabetes (12.2 %) and different fractures (11.3 %).

**Conclusions:**

We found that the main ailments for hospitalised patients were both chronic diseases like cardiovascular diseases, rheumatism, diabetes and acute conditions like fractures. The average hospital stay was 11 days.

**Key messages:**

• Understanding how causes of hospitalisation among elderly interact with the use of healthcare services might help to reduce risk factors associated with hospitalisation and improve the health systems.

• Further studies on hospital admissions among older persons with particular characteristics health conditions could help to reduce the burden of disease and improve the well - being of the elderly.

